# Effect of Universal Adhesive Etching Mode on Shear Bond Strength of Pulp Capping Materials to Deep Dentin

**DOI:** 10.1155/bmri/1496726

**Published:** 2025-07-09

**Authors:** Shahram Amirifar, Saba Tohidkhah, Seyedeh Mahsa Sheikh-Al-Eslamian, Mahdi Abbasi, Fatemeh Farshad, Elham Ahmadi

**Affiliations:** ^1^Department of Restorative Dentistry, School of Dentistry of Tehran University of Medical Sciences, Tehran, Iran; ^2^Minnesota Dental Research Center for Biomaterials and Biomechanics, School of Dentistry of University of Minnesota, Minneapolis, Minnesota, USA; ^3^Department of Population and Quantitative Health Sciences, Case Western Reserve University School of Medicine, Cleveland, Ohio, USA; ^4^Department of Operative Dentistry, School of Dentistry of Tehran University of Medical Sciences, Tehran, Iran; ^5^Dental Research Center, Dentistry Research Institute of Tehran University of Medical Sciences, Tehran, Iran

**Keywords:** dental bonding, dental pulp capping, shear strength

## Abstract

**Objective:** This study was aimed at investigating the shear bond strength (SBS) of composite to deep dentin with universal adhesive in total-etch (TE) and self-etch (SE) mode using different pulp capping materials.

**Methods:** One hundred twelve sound human third molars were divided according to universal adhesives (All-Bond Universal and G-Premio Bond), etching modes (TE and SE), and pulp capping materials (TheraCal PT, TheraCal LC, and Lime-Lite). After sectioning the tooth 2 mm above and 3 mm below the CEJ, the liner, adhesive, and composite were applied. Specimens were tested for SBS using the universal testing machine at a crosshead speed of 1 mm/min after restoring with composite. Data were analyzed with three-way ANOVA (*α* = 0.05).

**Results:** The highest and lowest SBS value was related to G-Premio bonding adhesive in SE mode using Lime-Lite (11.91 ± 0.77) and G-Premio bonding adhesive in SE mode using TheraCal PT (4.48 ± 0.83), respectively. Although the type of adhesive material did not have a significant impact on SBS values (*p* = 0.58), the mode of acid etching (*p* = 0.001) and the type of liner (*p* = 0.001) used were found to have a significant effect. Moreover, TE mode was associated with higher SBS values than SE mode.

**Conclusions:** Using TE mode increased the SBS; in contrast, liners reduced the SBS of the composite to deep dentin. Moreover, other generations of adhesives and comparison with universal bonding should be done in future studies.

## 1. Introduction

The preservation of the dentin–pulp complex in deep cavities is essential for maintaining the vitality of the pulp [1]. Pulp capping procedures involve the application of protective materials over exposed pulp tissue or deep pulp floor after the removal of carious tissue [2]. Calcium hydroxide has been a widely used cavity lining material for pulp capping treatments due to its antibacterial properties and ability to promote hard tissue formation, but its physical properties and solubility have limited its use over time [3].

Lime-Lite is a new alternative, a resin-based light-activated calcium hydroxide–based material that can be used as a liner and base [4]; it contains hydroxyapatite (HA) and releases hydroxyl, fluoride, and calcium [5]. Additionally, tricalcium silicate–based cements, including mineral trioxide aggregate (MTA), are a viable alternative to calcium hydroxide for treating deep cavities [6].

TheraCal LC (Bisco Inc., Schamburg, Illinois, United States) and TheraCal PT (Bisco Inc., Schamburg, Illinois, United States) are recent examples of light-curable and dual-cured, resin-modified calcium silicate–based materials, respectively, designed for use in vital pulp treatments. They possess easy handling properties and greater release of calcium ions compared to MTA and Dycal [7, 8]. However, their biological properties are still under investigation, and their use as pulp capping materials is yet to be fully recommended [6].

The bond strength between the liner–resin composite complex and dentin is crucial for restoration longevity and pulp vitality. Changes to pulp capping material, such as those caused by etching procedures used prior to the application of resin composite restorative materials [9], may impact the bond strength, emphasizing the importance of adhesive selection.

Recently, universal adhesives have been introduced as a versatile option for bonding restorative materials to dentin. Unlike traditional etch-and-rinse adhesives, they can be used in different ways depending on the clinical situation and the clinician's preference. The differences between universal adhesives and traditional adhesives include the initial pH, type of acidic monomer, concentration of water and solvents, and the hydrophilicity of the bonding layer [10]. It is important to note that the pH and solubility of the adhesive can impact its bonding effectiveness, with lower pH and higher solubility leading to decreased bond strength [11].

Some studies have reported that the internal adaptation [12, 13] and shear bond strength (SBS) [3] of resin composite restorations are more favorable in cases where no liner is applied. However, the use of liners is still necessary in certain situations.

We selected the SBS test because it is a widely accepted, simple, and reproducible method for assessing the adhesion between dentin and restorative materials. It effectively simulates the clinical stresses at the dentin–liner interface, making it suitable for evaluating the impact of bioactive [12].

Moreover, it evaluates the effect of bioactive liner materials on the adhesion between the composite-liner complex and dentin, especially when different bonding agents are used. While numerous studies have assessed general bond strength, the influence of new bioactive photopolymerizable liners—such as TheraCal PT, which features a dual-curing mechanism—on the composite–dentin bond has not been extensively investigated. Therefore, our aim was to better understand how these liners, in combination with various bonding agents, affect the overall adhesive interface, justifying the use of the shear test in this context. Given the limited information available in the literature regarding the behavior of lining materials beneath composite restorations, the present study was aimed at investigating the SBS of composite to deep dentin with universal adhesive in total-etch (TE) and self-etch (SE) mode using different pulp capping materials.

## 2. Materials and Methods

This in vitro experimental study was conducted at the Tehran University of Medical Sciences Dental Research Center. The study was conducted according to the guidelines of the Declaration of Helsinki and approved by the Ethics Committee of the Tehran University of Medical Sciences Ethical Committee (No. IR.TUMS.DENTISTRY.REC.1400.148).

Based on a prior research study utilizing the same methodology [3], 112 sound human third molars extracted within the past 3 months were selected and examined using a stereomicroscope (SZX 16; Olympus, Tokyo, Japan) to ensure they were free of fractures, restorations, caries, calculus, and any other abnormalities.

Then, they were disinfected using a 0.5% chloramine T solution for 1 week at 4°C. Subsequently, during the experimental procedures, the specimens were kept in distilled water at 37°C.

The samples were prepared by utilizing a high-speed D&Z diamond disc with water coolant to have a disc-shaped section of the tooth (2 mm above and 3 mm below the cemento-enamel junction (CEJ)). To ensure a uniform thickness of 5 mm, discs were measured with a digital caliper (Mitutoyo, Japan). Then, the smear layer was standardized by using a polishing machine equipped with 600-grit sandpaper and water as a lubricant.

The polyethylene tube was used to standardize the dimensions of the liners (1 mm height and 2 mm internal diameter) and the resin composite restorative materials (4 mm height and 5 mm internal diameter).

Then, all teeth were assigned to four groups (three experimental and one control) using a stratified randomization method.

**Figure 1:**
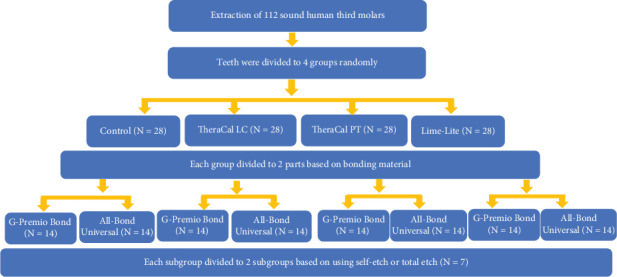
Schematic illustrations of the procedure of this study.

In all groups, All-Bond Universal (Bisco Inc., Schaumberg, Illinois, United States) with a pH of 3.3 or G-Premio Bond (GC, Tokyo, Japan) with a pH of 1.5 was used in different TE and SE modes. In the experimental groups, following the application of liners including TheraCal LC, TheraCal PT (Bisco, Schaumburg, Illinois, United States), and Lime-Lite (Pulpdent Corporation, Watertown, Massachusetts, United States) in each test group, the dentin surfaces and the liners in the TE and SE groups were treated according to the manufacturer's instructions (Table 1 and Figure 1).

The polyethylene tube (4 × 5 mm) was then placed over the lining material, filled with the resin composite (Z250, 3M ESPE, St. Paul, Minnesota, United States) using the incremental technique (two 2-mm increments), and cured with a curing unit for 40 s.

In all groups, the bonding agent and restorative materials were cured by a light-curing unit (Woodpecker, DTE LUX, China) (1000 mW/cm^2^) at a standardized distance of 1 mm. Finally, the polyethylene tubes were removed using a sharp blade.

To equivalent to 6 months of oral function, the samples underwent thermocycling (Delta Tpo2, Nemo, Mashhad, Iran) consisting of 5000 cycles of alternating temperatures between 5°C and 55°C with a dwell time and transfer time of 20 s.

The SBS of the specimens was measured using a universal testing machine (Zwick/Roell Z050, Ulm, Germany) that employed a 50-kg load cell at a crosshead speed of 1 mm/min until the point of bond failure was reached.

Then, the test specimens were examined through a stereomicroscope (SZX 16; Olympus, Tokyo, Japan) with a magnification of 25x to determine the nature of the failure. The modes of failure were categorized into three groups, namely, cohesive (the fracture being entirely within the liner or resin composite), adhesive (the fracture occurring at the interface of the material and dentin with less than 10% of the liner remaining on the dentin), and mixed (combination of both cohesive and adhesive failure) [13].

The data was analyzed using the Statistical Package for Social Sciences (SPSS) Version 25. The mean and standard deviation of the bond strength of composite to deep dentin under different liner conditions, etching modes, and bonding agents were calculated and reported (Table 2). The effects of the type of adhesive, etching modes, and liner type on the bond strength values were statistically evaluated using a three-way analysis of variance (ANOVA) test. The bond strength values were compared among subgroups using independent *t*-test, one-way ANOVA, and Tukey's and Games–Howell post hoc tests. The significance level (*α*) was set at 0.05 in this study.

## 3. Results

The type of adhesive material did not have a significant impact on SBS values (*p* = 0.58); however, the mode of acid etching and the type of liner used were found to have a significant effect (*p* < 0.001) (Table 3).

The highest SBS value was related to G-Premio universal adhesive in SE mode using Lime-Lite liner (11.91 ± 0.77), and the lowest was related to G-Premio universal adhesive in SE mode using TheraCal PT liner (4.48 ± 0.83) (Table 2). Furthermore, in the control groups, the SBS values in the TE pattern were significantly higher than those obtained in the SE pattern using both All-Bond Universal (*p* = 0.05) and G-Premio universal adhesives (*p* = 0.03).

Moreover, the SBS values obtained from the application of the TheraCal PT liner with the All-Bond Universal adhesive in both TE and SE patterns were significantly higher when compared to those obtained from the use of the G-Premio universal adhesive (*p* = 0.03 and *p* = 0.04, respectively). In addition, there is no significant difference in the bond strength of Lime-Lite in both etching modes or adhesive differences (*p* > 0.05). Finally, the TheraCal LC group showed significantly higher SBS when the All-Bond Universal adhesive was applied using the TE pattern, compared to the SE pattern (*p* = 0.01).

**Figure 2:**
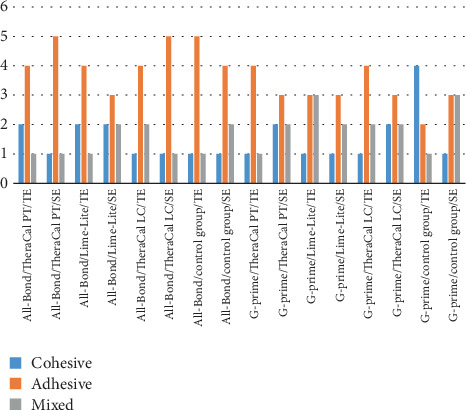
Graphical illustration of failure modes. TE, total-etch; SE, self-etch.

The distribution of different modes of failure in each group of All-Bond Universal adhesive and G-Premio Bond Universal adhesive reveals that, in most groups, adhesive failure was more prevalent than cohesive and mixed failure, except for the control group using the G-Premio Bond Universal adhesive in the TE pattern, where cohesive failure was more frequent than the other two modes of failure. The type of liner material also influenced the fracture modes, with TheraCal PT displaying a higher proportion of adhesive fractures compared to TheraCal LC and Lime-Lite (Figure 2).

## 4. Discussion

When pulp capping materials fail to properly adhere or adapt to dentin, gaps can form, causing fluid fluctuations in dentinal tubules that stimulate pain receptors in the pulp tissue and lead to postoperative sensitivity and pain [2]. The present study illustrated that using TE mode in both cases of using liners and not using them increased the SBS. In addition, the use of liners reduced the SBS of the composite to deep dentin compared to the case where the liner was not used.

The technique of adhesive application plays a more significant role in bond strength than the type of composite or application method [14]. Hydrophilic monomers and increased solvent content have simplified adhesive systems, reducing technical sensitivity, saving time, and being easy to use. Universal adhesives combine acidic primers and adhesives into one solution, enabling direct or indirect restoration of enamel and dentin in a “one-bottle” form. These adhesives differ from etch-and-rinse adhesives based on initial pH, acidic monomer type, water and solvent concentration, and bonding layer hydrophilicity and can be classified as ultramild (pH > 2.5), mild (pH = 2.5), or strong systems (pH < 1.5) [15].

To simulate the oral environment and achieve long-term bond strength in vitro, researchers have utilized various aging methods, including water immersion, mechanical cycles, and/or thermocycling [16]. Thermocycling is preferred as it can mimic the repetitive expansion and contraction stresses caused by temperature fluctuations within the oral cavity. It has been suggested that 10,000 cycles are equivalent to 1 year of clinical service, assuming 20–30 cycles per day intraorally [17]. In this study, we utilized 5000 cycles, corresponding to 6 months of oral cavity exposure [18]. Moreover, in line with the present study, the study that used 1500 cycles in 1 day demonstrated that using etch before adhesive resulted in the highest bond strength to fast-setting calcium silicate cement [19].

In the present study, the control group of both universal adhesive systems exhibited significantly higher bond strength in the TE pattern compared to the SE pattern. However, the recent study conducted on MTA demonstrated the highest bond strength with the two-step SE group at 15 min, and the calcium-enriched mixture exhibited the highest bond strength with the two-step SE groups at the 72-h interval. This controversy may be due to the difference in materials and duration of follow-up [20].

Although the use of phosphoric acid before the application of universal adhesive is not in the instruction, a potential chemical interaction between MDP and demineralized dentin has been found in some studies [21]. Previous studies have investigated the bond strength of resin composite to dentin when various pulp capping materials were used [2, 3]. For instance, a study illustrated that the annual failure rate between nanohybrid composite and a flowable composite for the cavity lining led to significant differences, which caused higher failure in the group containing the liner [22]. However, these studies have mostly utilized Single Bond Universal adhesive with only the SE mode. In contrast, we used two brands of universal adhesives (All-Bond Universal and G-Premio Bond) in TE and SE etching modes.

Furthermore, there is no prior publication that has assessed the bond strength of resin composite to dentin in the presence of TheraCal PT as a pulp capping material, with any type of adhesive material. In the present study, TheraCal PT showed the lowest SBS value in SE mode when G-Premio Bond was used. TheraCal PT has an almost neutral pH, which may result in the production of more porosity when a bonding agent with a pH lower than 1.5 is applied, and this porosity may not be adequately filled by the adhesive [23]. These results are in accordance with those obtained by Yavuz et al. [24], who compared the interaction between dentin and three pulp capping materials (NeoPutty, TheraCal PT, and Biodentin) using Single Bond Universal in the SE mode. TheraCal PT showed significantly higher microleakage and larger microgap width between dentin and the pulp capping material in SEM compared to NeoPutty and Biodentin. However, there is some lack of explanation regarding the standardization of pulp capping material thickness and the potentially influencing effects of the destructive SEM sample preparation in the previous study. The increased gap in TheraCal PT is attributed to the polymerization shrinkage of its resin components compared to the other two materials [24].

Lime-Lite exhibited the highest SBS values in SE mode using G-Premio Bond. It also reported that no significant difference was observed in the bond strength of Lime-Lite among any of the study groups or the control group, which suggests that the etching modes and adhesive type did not significantly affect its bond strength. This could be attributed to its low solubility in water and acetic acid [25]. Additionally, the manufacturer claims that Lime-Lite contains HA and releases calcium, fluoride, and hydroxyl ions [5]. Thus, the presence of HA may have contributed to enhancing the bond quality in this group.

Our study revealed that the TheraCal LC group showed significantly higher SBS when the All-Bond Universal adhesive was applied using the TE pattern, compared to the SE pattern. This difference could be attributed to the lack of washing of the materials released on the liner surface after applying the adhesive.

The analysis of the modes of fracture in relation to etching type, adhesive type, and liner material provides valuable insights into the performance and potential implications of these factors. The distribution of fracture modes was found to be similar for both TE and SE techniques, with adhesive fractures being the most common mode in both cases. This suggests that the etching type may not significantly affect the fracture mode. The analysis of adhesive types revealed a higher prevalence of adhesive fractures with All-Bond adhesive, irrespective of the etching type or liner material. In contrast, G-Premio adhesive exhibited a higher proportion of cohesive fractures, indicating that the adhesive properties of different bonding agents can influence the fracture mode, with All-Bond potentially offering stronger adhesion at the interface. The type of liner material also influenced the fracture modes, with TheraCal PT displaying a higher proportion of adhesive fractures compared to TheraCal LC and Lime-Lite. This difference may be due to variations in the physical properties and bonding characteristics of the liner materials, suggesting that TheraCal PT may provide a less robust bond to the dentin, resulting in a higher incidence of adhesive failures.

This study demonstrates the impact of adhesive type, acidity, and etching modes on bond strength, providing valuable guidance for clinicians in adhesive system selection and application. Additionally, it provides insights into the behavior of specific pulp capping materials (TheraCal PT, TheraCal LC, and Lime-Lite) regarding their bond strength to dentin, aiding clinicians in material selection and anticipating potential limitations. These results provide a foundation for further research and should be interpreted in the context of in vitro conditions. Clinical validation is required to fully understand the behavior of these materials in the oral cavity.

## 5. Conclusion

Within the limitation of this study, it can be concluded that using TE mode increased the SBS; in contrast, liners reduced the SBS of the composite to deep dentin. Moreover, other generations of adhesives and comparison with universal bonding should be done in future studies.

## Figures and Tables

**Table 1:** Materials used in this study and their application instruction.

**Materials**	**Type and composition**	**Application instruction**
All-Bond Universal (Bisco, Schaumburg, Illinois, United States)	Ultramild Universal adhesive pH: 3.3 2-HEMA, 10-MDP, Bis-GMA, ethanol, water, initiators, pH = 3.2	Total-etch mode: Dentin surface was conditioned with phosphoric acid. Conditioned surface was rinsed with water for 15 s (three-way dental syringe) and air-dried. Two separate coats of adhesive are applied, with rubbing action for 10–15 s per coat. No light cure between coats. Gentle stream of air applied over the liquid for at least 10 s. Light irradiates for 10 s. Self-etch mode: Phosphoric acid pre-etching was not performed.
G-Premio Bond (GC Corp., Tokyo, Japan)	Strong Intermediate universal adhesive pH: 1.5 10-MDP, 4-MET, MEPS, methacrylate monomer, acetone, water, silica, initiators, pH = 1.5	Total-etch mode: Dentin surface was conditioned with phosphoric acid. Conditioned surface was rinsed with water for 15 s (three-way dental syringe) and air-dried. One coat of adhesive is applied. The adhesive is left undisturbed for 5–10 s. Air dry under maximum pressure for 5 s. Light irradiates for 10 s. Self-etch mode: Phosphoric acid pre-etching was not performed.
TheraCal LC (Bisco, Schaumburg, Illinois, United States)	Pulp cap agent Portland cement Type III polyethylene glycol dimethacrylate, barium zirconate	1- Apply to operatory area of the preparation. 2- Light cure for 20 s. 3- Apply the adhesive agent.
TheraCal PT (Bisco, Schaumburg, Illinois, United States)	Pulp cap agent Base: Silicate glass-mix cement, polyethylene glycol dimethacrylate, BisGMA, barium zirconate Catalyst: Barium zirconate, ytterbium fluoride, initiator	
Lime-Lite (Pulpdent Corporation, Watertown, Massachusetts, United States)	Pulp cap agent Calcium hydroxy phosphate (hydroxyapatite), urethane dimethacrylate resin, barium sulfate, fluoride salt, photoinitiator	
FiltekZ250 (3MESPE, St. Paul, Minnesota, United States)	Nanohybrid resin composite Bis-GMA, UDMA, Bis-EMA, zirconia/silica filler (without silane treatment)	1.5–2 mm thickness of composite layer applied on dentin surface and cure for 20–40 s.

**Table 2:** The mean ± SD of the shear bond strength (SBS) values obtained through the use of different bonding agents with different liners in E (TE) and self-etching (SE) modes.

**Adhesive**	**Type of liner**	**Etching mode**	**M** **e** **a** **n** **SBS** ± **SD**
All-Bond Universal	TheraCal PT	TE	10.97 ± 4.33
		SE	8.14 ± 3.82
	TheraCal LC	TE	11.18 ± 5.06
		SE	4.63 ± 1.44
	Lime-Lite	TE	11.11 ± 3.22
		SE	8.11 ± 4.24
	Control	TE	14.17 ± 4.62
		SE	8.44 ± 1.85

G-Premio Bond	TheraCal PT	TE	5.49 ± 4.17
		SE	4.48 ± 0.83
	TheraCal LC	TE	7.98 ± 2.46
		SE	6.57 ± 1.69
	Lime-Lite	TE	10.18 ± 1.85
		SE	11.91 ± 0.77
	Control	TE	16.43 ± 1.41
		SE	10.86 ± 4.2

Abbreviations: SBS, shear bond strength; SD, standard deviation; SE, self-etching; TE, total-etch.

**Table 3:** The effects of liner type, bonding, and etching pattern along with their interaction effects on the shear bond strength of composite with deep dentin by three-way analysis of variance test.

**Variable**	**p** ** value**
Adhesive	0.58
Etching mode	0.001
Liner	0.001
Adhesive and etching mode	0.02
Adhesive and liner	0.001
Etching mode and liner	0.04
Adhesive and etching mode and liner	0.49

## Data Availability

The data that support the findings of this study are available from the corresponding author upon reasonable request.
